# Supplementary material for: The Neuroregenerative Effects of IncobotulinumtoxinA (Inco/A) in a Nerve Lesion Model of the Rat
Source: Int J Mol Sci. 2025 Aug 2;26(15):7482. doi: 10.3390/ijms26157482 (PMC12347775; doi:10.3390/ijms26157482)
Supplement: Supplementary file 1 [file ijms-26-07482-s001.zip › ijms-3767559-supplementary.pdf]

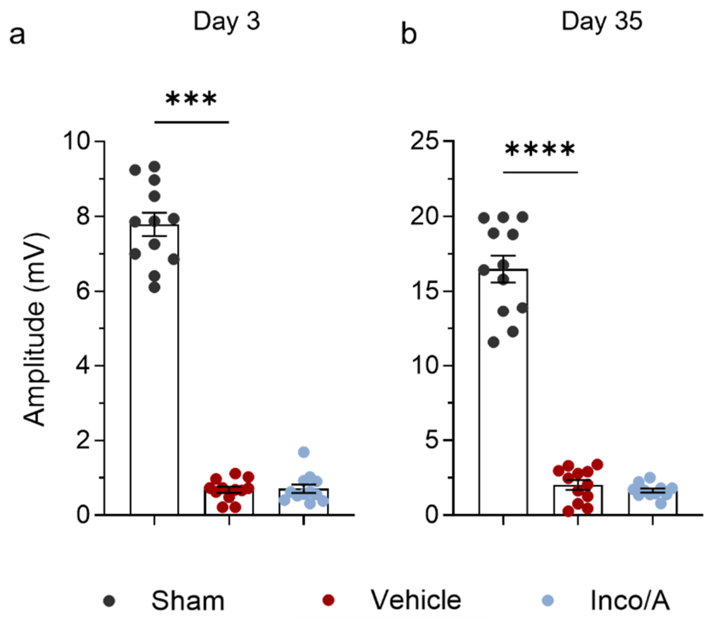

Supp. Figure S1. Rats showed a reduced AP amplitude after CCI measured with CMAP. Histograms showing amplitude of CMAP recordings on day 3 (a) and day 35 (b) post CCI. Note that CCI decreases amplitude values suggesting nerve damage (For Day 3, Kruskal-Wallis test, ****P*<0.001; for day 35, One-Way ANOVA test, *****P*<0.0001). Each dot represents the value of an individual animal (n=12 for each group). One outlier was discarded for the analysis according to Grubb`s outlier analysis (Day 35, Inco/A group).

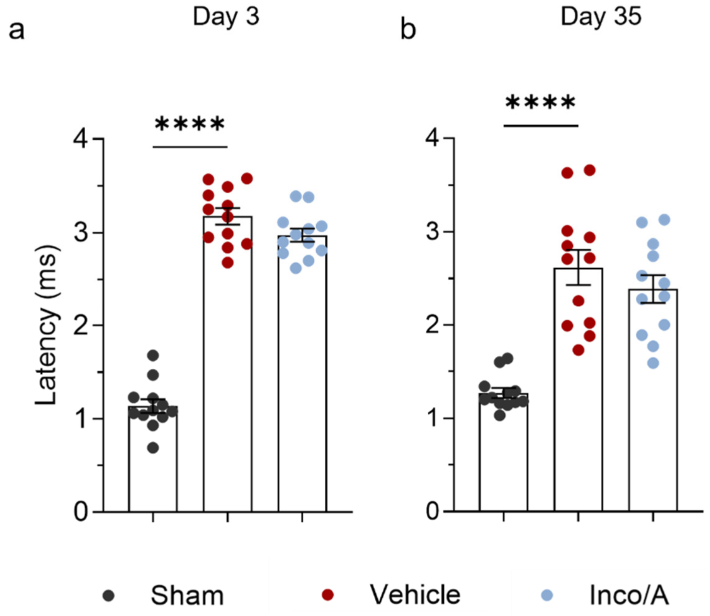

Supp. Figure S2. Rats showed reduced AP latencies after CCI measured with CMAP. Histograms showing delayed AP latencies in AP measured with CMAP recordings at day 3 (a) and day 35 (b) post CCI. Note that CCI delays AP latencies suggesting nerve damage (One-Way ANOVA test; ****P<0.0001). Each dot represents the value of an individual animal (n=12 for each group).

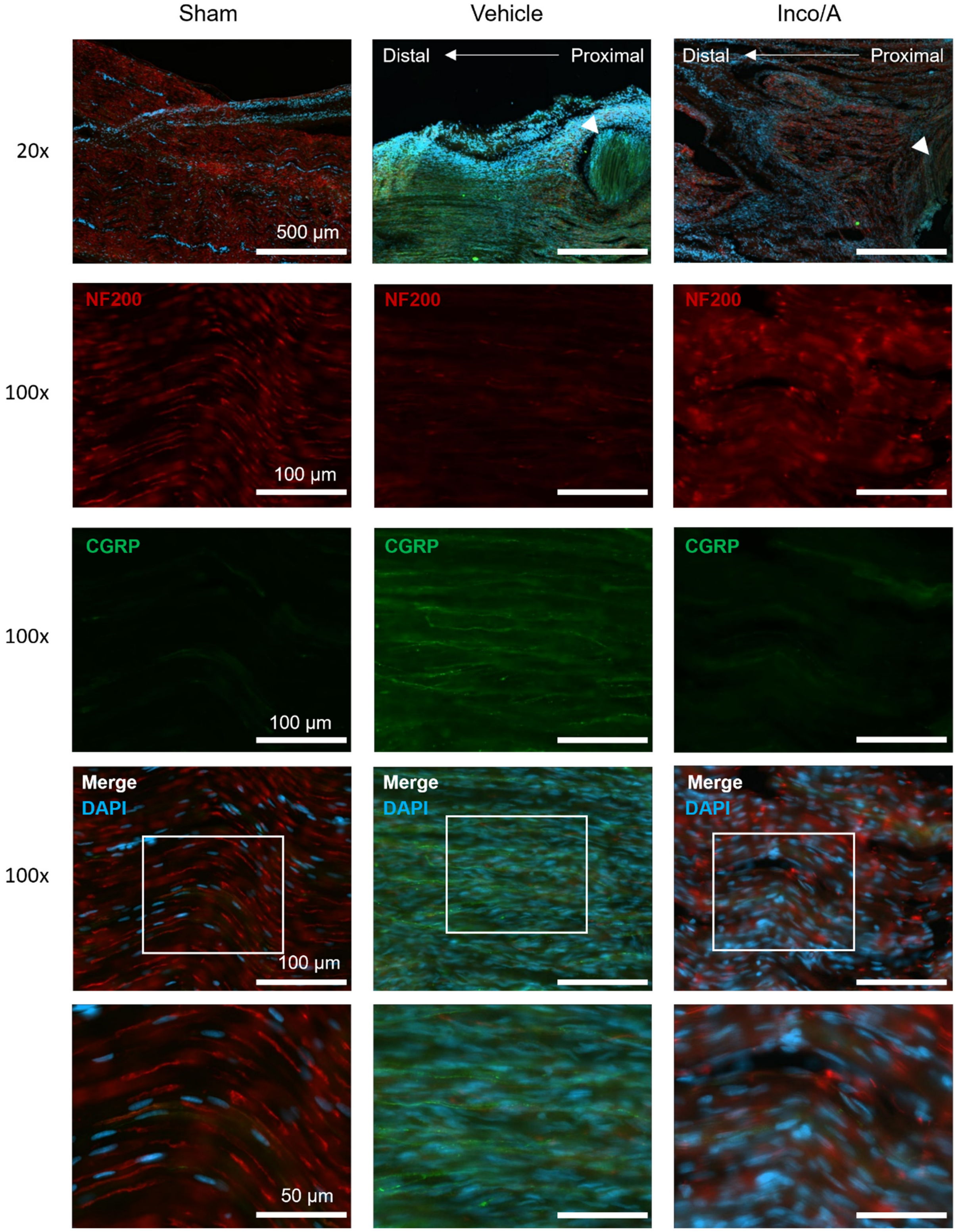

Supp Figure S3: Following nerve injury, treatment with Inco/A in rat led to the restoration of myelinated fibers and a reduction in CGRP levels in the sciatic nerves. Immunostainings at different magnifications showing NF200 and CGRP expression in longitudinal sections in sciatic nerves from sham, vehicle-treated and Inco/A-treated animals at day 56

post-CCI. Proximal and Distal directions with respect to the injury site are indicated. The white arrowhead indicated the injury site.

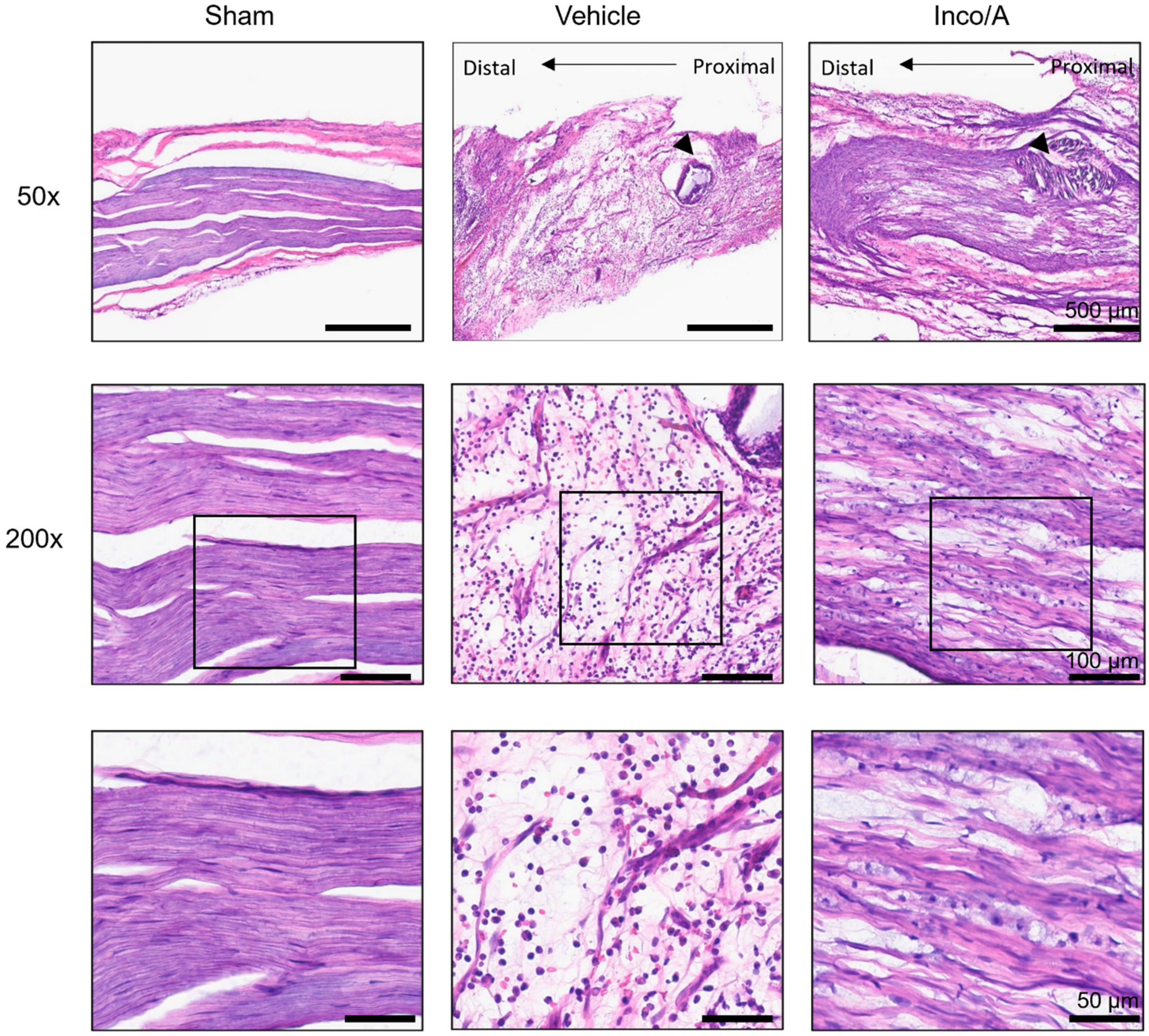

Supp. Figure S4: Inco/A attenuated inflammation in the injured sciatic nerve. Longitudinal sections at different magnifications of sciatic nerves stained with Hematoxylin from sham, vehicle- and Inco/A-treated animals 56 days post-CCI. Proximal and Distal directions with respect to the injury site are indicated. The black arrowhead indicated the injury site.

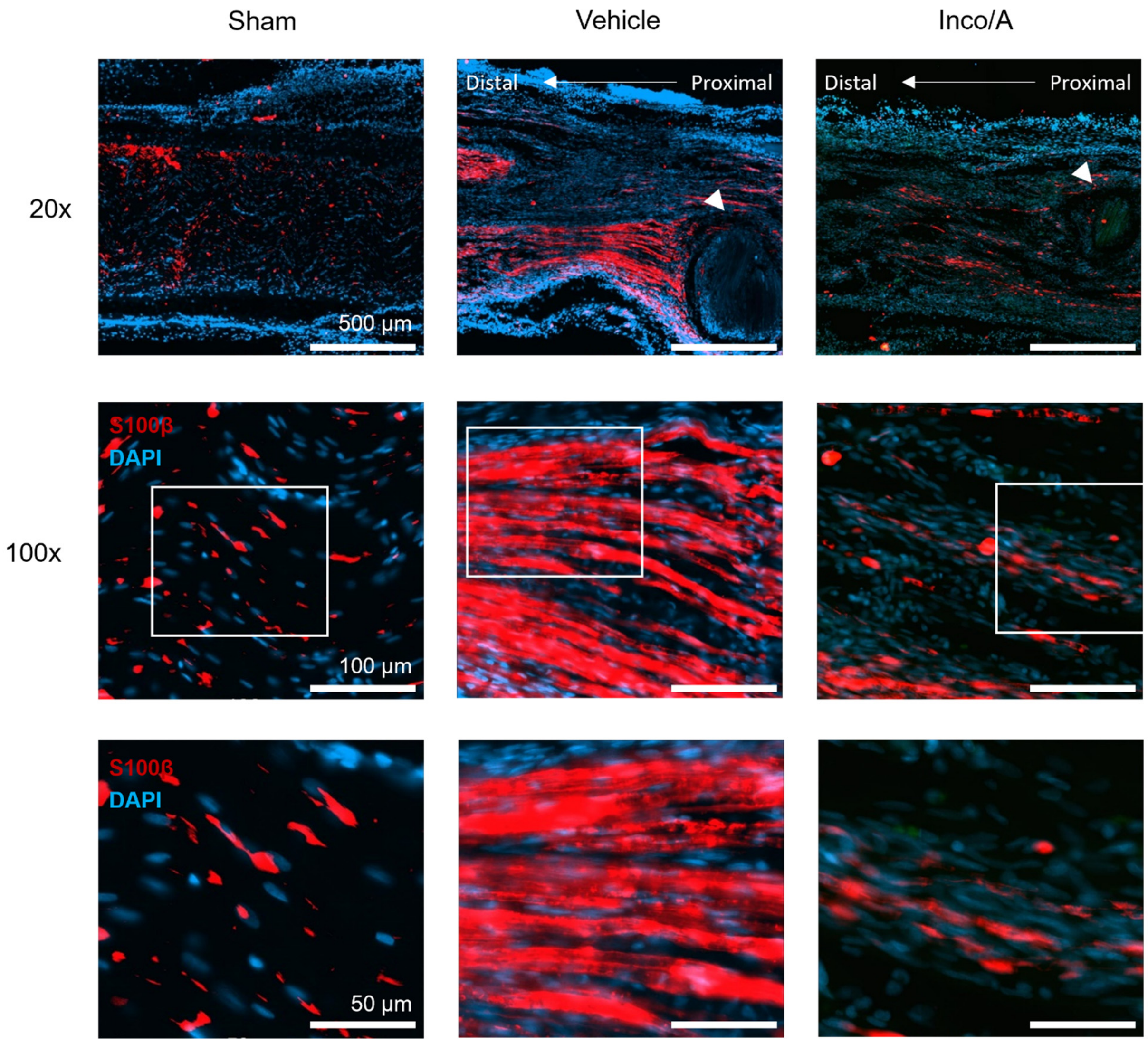

Supp. Figure S5: Inco/A counteracted Schwann cell migration and proliferation into the sciatic nerve following CCI. Immunostainings at different magnifications for S100β in longitudinal sections in sciatic nerves from sham, vehicle-treated and Inco/A-treated animals at day 56 post-CCI. Proximal and Distal directions with respect to the injury site are indicated. The white arrowhead indicated the injury site.

Supp. Table S1. Comparison of nerve regeneration studies in various animal and nerve injury models. *CCI,* chronic constriction injury; *SNC,* sciatic nerve crush; *U*, units; *Kg*, kilogram. *NA,* not available; *Approx*., approximately.

	**BoNT/A formulation**	**Timing of injection**	**Dose**	**Delivery**	**Strains and nerve injury model**
**Marinelli,** ***et al.*** **(2010)**	150-kDa; di-chain molecular of purified BoNT/A	Day 6 after CCI	Mice: max. 7-8 U/Kg<br><br>Rats: max. 4-5 U/Kg (approx. 1.6 U/animal)	Single Intraplanar application	CD1 male mice and male Wistar rats / CCI
**Cobianchi,** ***et al.*** **(2017)**	Purified version of BoNT/A	Day 0, immediately after sciatic nerve crush	Mice: max. 7-8 U/Kg	Single application.<br><br>Intraneural for SNC ; Intraplanar for CCI	Female C575BL/6 mice. / SNC and CCI
**Hwang,** ***et al.*** **(2023)**	NA	Day 0, immediately after nerve crushing injury	3.5, 7 and 14 U/Kg (approx. 0.6, 1, 1.4 and 2.8 U/animal)	Single intraneural application	SPF Sprague Dawley rats / SNC
**Seo,** ***et al*** **(2024)**	Onabotulinum toxin A (BOTOX®, Allergan)	One group on day 0, immediately after nerve-crushing injury.<br><br>One group one week after nerve injury.	7 U/Kg (approx. 1.4 U/animal)	Perineurally	SPF Sprague Dawley rats / SNC
**Current study**	IncobotulinumtoxinA (Xeomin®, Merz Therapeutics)	Day 0, immediately after nerve injury with a second dose at day 21 post surgery	4 U	Perineurally	Male Sprague Dawley rats / CCI
